# Identification of risk model based on glycolysis-related genes in the metastasis of osteosarcoma

**DOI:** 10.3389/fendo.2022.1047433

**Published:** 2022-10-27

**Authors:** Wei Huang, Yingqi Xiao, Hongwei Wang, Guanghui Chen, Kaixiang Li

**Affiliations:** ^1^ Department of Orthopaedics, Dongguan Tungwah Hospital, Dongguan, Guangdong, China; ^2^ Department of Pulmonary and Critical Care Medicine, Dongguan Tungwah Hospital, Dongguan, Guangdong, China

**Keywords:** Osteosarcoma, glycolysis, prognostic risk model, machine learning, INSR

## Abstract

**Background:**

Glycolytic metabolic pathway has been confirmed to play a vital role in the proliferation, survival, and migration of malignant tumors, but the relationship between glycolytic pathway-related genes and osteosarcoma (OS) metastasis and prognosis remain unclear.

**Methods:**

We performed Gene set enrichment analysis (GSEA) on the osteosarcoma dataset in the TARGET database to explore differences in glycolysis-related pathway gene sets between primary osteosarcoma (without other organ metastases) and metastatic osteosarcoma patient samples, as well as glycolytic pathway gene set gene difference analysis. Then, we extracted OS data from the TCGA database and used Cox proportional risk regression to identify prognosis-associated glycolytic genes to establish a risk model. Further, the validity of the risk model was confirmed using the GEO database dataset. Finally, we further screened OS metastasis-related genes based on machine learning. We selected the genes with the highest clinical metastasis-related importance as representative genes for *in vitro* experimental validation.

**Results:**

Using the TARGET osteosarcoma dataset, we identified 5 glycolysis-related pathway gene sets that were significantly different in metastatic and non-metastatic osteosarcoma patient samples and identified 29 prognostically relevant genes. Next, we used multivariate Cox regression to determine the inclusion of 13 genes (ADH5, DCN, G6PD, etc.) to construct a prognostic risk score model to predict 1- (AUC=0.959), 3- (AUC=0.899), and 5-year (AUC=0.895) survival under the curve. Ultimately, the KM curves pooled into the datasets GSE21257 and GSE39055 also confirmed the validity of the prognostic risk model, with a statistically significant difference in overall survival between the low- and high-risk groups (P<0.05). In addition, machine learning identified INSR as the gene with the highest importance for OS metastasis, and the transwell assay verified that INSR significantly promoted OS cell metastasis.

**Conclusions:**

A risk model based on seven glycolytic genes (INSR, FAM162A, GLCE, ADH5, G6PD, SDC3, HS2ST1) can effectively evaluate the prognosis of osteosarcoma, and *in vitro* experiments also confirmed the important role of INSR in promoting OS migration.

## 1 Introduction

Osteosarcoma (OS) is adolescents’ most common primary bone malignant tumor, accounting for about 20-34% of all primary malignant bone tumors (1). Although OS tumors can be improved by complete surgical resection and chemotherapy, their prone to recurrence, and metastasis often lead to a poor prognosis for OS patients (2). Even after long-term standardized chemotherapy, OS still has a 35% recurrence rate (3). Metastasis remains the leading cause of death in OS patients, with the major metastases being in the lungs, other bone tissue, and lymph (4, 5). However, up to 80-90% of OS patients with metastatic cancer are difficult to detect clinically due to the small size of early metastases and the low sensitivity of diagnostic imaging (6). Patients with OS with combined metastases usually have a poor prognosis, with only about 20% 5-year survival rate, so there is an urgent need for more sensitive screening methods to identify early metastases in OS (7).

More and more studies have shown that glycolysis-related genes are closely related to tumor occurrence, metastasis and prognosis (8). Glycolysis is the main pathway of glucose metabolism in cancer cells. Cancer cells can undergo glycolysis to metabolize glucose to lactic acid instead of oxidative phosphorylation (OXPHOS), which is the Warburg effect (9). The metabolic shift from OXPHOS to glycolysis is often considered a sign of OS (10). Especially under hypoxic conditions, the invasive potential of OS cells is increased; the angiogenesis, poor chemotherapy response and overall survival rate of OS animals are also significantly reduced (9). The characteristics of the unlimited proliferation of tumor cells make the cells often in a state of hypoxia. The glycolysis pathway can improve the tolerance of tissue cells to hypoxia to avoid apoptosis induced by OXPHOS. At the same time, excessive lactic acid produced by the glycolysis pathway can also decompose and destroy the cell matrix around tumor cells, thus promoting the migration of tumor cells to other tissues and organs.

At present, the prognosis of OS still lacks effective prediction methods, and the relationship with genes related to the glycolysis pathway is still unclear. We hope to use bioinformatics methods to explore the relationship between glycolytic pathway-related genes and the metastasis and prognosis of patients with OS and construct a prognostic risk model for OS, to provide a reference for the survival assessment of OS patients.

## 2 Materials and methods

**Figure 1:**
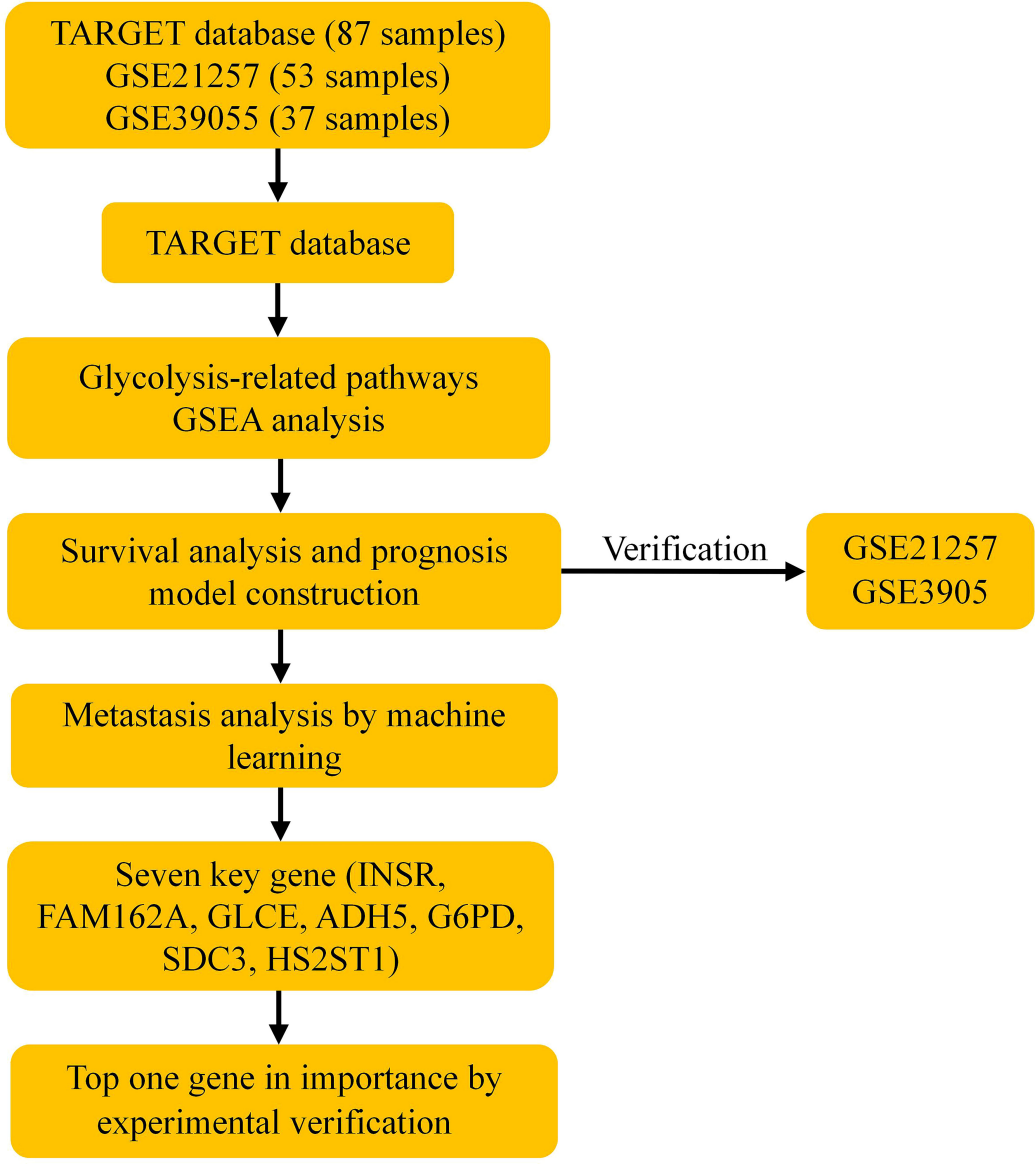
Schematic workflow of a risk model for osteosarcoma prognosis constructed based on glycolysis-related genes. GSEA, Gene set enrichment analysis.

The processing flow of this study is shown in **Figure 1**.

### 2.1 OS patient data set to download and standardized processing

The clinical information and mRNA sequencing data of OS patients were downloaded through the TARGET database (https://ocg.cancer.gov/programs/target), and 1. Patients with missing survival information were eliminated, and 87 patients were included. At the same time, the data sets GSE21257 and GSE39055 were obtained from Gene Expression Omnibus (GEO, https://www.ncbi.nlm.nih.gov/geo/). Finally, we use the R 4.0.2 limma software to perform log2 standardization on the mRNA data of the OS samples and take the average value of genes with multiple probes. The specific information of the included data set is shown in **Table 1**. See Supplementary Material for all data sets.

**Table 1:** Basic characteristics of the gene expression profile data.

Dataset	Platform	Normal	Tumor
TARGET	Illumina HiSeq	0	87
GSE21257	GPL10295 Illumina human-6 v2.0 expression beadchip	0	53
GSE39055	GPL14951 Illumina HumanHT-12 WG-DASL V4.0 R2 expression beadchip	0	37

### 2.2 Gene set enrichment analysis (GSEA)

First, we use GSEA (http://software.broadinstitute.org/gsea/index.jsp) to determine the glycolysis-related pathway gene set from the Molecular Signatures Database (MSigDB, https://www.gsea-msigdb.org/gsea/msigdb/). Then GSEA was also performed on the osteosarcoma data set of the TARGET database to explore the differences between the glycolysis-related pathway gene sets in the samples of patients with metastatic and non-metastatic osteosarcoma. P<0.05 is set as the critical value.

### 2.3 Screening of OS prognostic genes and construction of risk models

We obtained 326 human glycolysis-related genes through the glycolysis-related pathway gene set provided by the MSigDB database and extracted human glycolysis-related genes from the osteosarcoma dataset in the TARGET database. Next, we use the survival package of R language to perform a univariate Cox regression analysis and screen out glycolytic genes that are significantly related to the overall survival of patients with OS (P<0.05). Then, through multivariate Cox regression analysis, we screened for independent prognostic genes, constructed a patient prognostic risk model, and drawn a nomogram.

The Risk Score=expmRNA1×β1+expmRNA2×β2+……+expmRNAn×βn (exp:The expression level;β is the regression coefficient of multivariate Cox regression analysis).

### 2.4 Analysis of Gene Ontology (GO) and Kyoto Encyclopedia of Genes and Genomes (KEGG) pathways

The R language cluster profile package for survival-related genes is used for GO and KEGG pathway enrichment analysis. Upload survival-related genes to the STRING database (https://string-db.org/) to construct protein-protein interactions (PPI).

### 2.5 OS risk model predictive value evaluation and verification

Then, the risk score of OS patients was calculated through the constructed prognostic risk model. The OS patients from the TARGET database were divided into high-risk and low-risk groups based on the median value; the R software survival and survminer packages were used to draw Kaplan-Meier (K-M) curves and receiver operating characteristic (ROC) curves to evaluate the predictive value of the prognostic model; for the data set GSE21257 and GSE39055 also draw the K-M curve based on the prognostic risk model.

### 2.6 Further screening for OS metastasis-related genes based on machine learning

Beside, machine learning is also an important prognostic gene screening method, referring to previous research (4, 5, 11). Next, a support vector machine (SVM) and random forest (RF) were used to construct a specimen classification model to screen the most closely related prognostic genes for OS metastasis. Hierarchical clustering analysis and unsupervised clustering methods were performed for the TARGET dataset based on the expression values of prognostic genes. In addition, the performance of different types of samples is evaluated by iterating combinations of random features until the optimal variety of features is obtained. Ultimately, representative genes are screened that can be used as representative genes for clinical metastasis relevance.

### 2.7 *In vitro* experimental validation

The INSR gene with the highest importance in tumor metastasis-related aspects obtained by the RF classifier algorithm was selected as a representative gene for *in vitro* experimental validation.

#### 2.7.1 Cell culture

MG-63 osteosarcoma cell line (ATCC, CAS cell bank) and hFOB1.19 osteoblast cell line (ATCC, CAS cell bank) were selected for *in vitro* validation. The cells were cultured in a DMEM medium (10% FBS and 1% penicillin/streptomycin) at 37°C and 5% CO_2_ in an incubator according to previous culture methods (12).

#### 2.7.2 Western blotting (WB)

First, total protein was extracted from MG-63 cells and hFOB1.19 cells using RIPA buffer. Next, complete proteins were separated by 10% SDS-PAGE and transferred to polyvinylidene fluoride (PVDF) membranes. The membranes were next closed with 5% skim milk powder in TBST at 37°C for 1h and incubated with primary antibodies at 4°C overnight. The antibodies were anti-INSR antibody (#38126, Signalway Antibody LLC, SAB, USA), anti-GAPDH antibody (#23001, Signalway Antibody LLC, SAB, USA) antibody as an internal reference. Then TBST was washed for 20 minutes and incubated with anti-rabbit horseradish peroxidase-conjugated secondary antibody for 1 hour at 37°C. Finally, the protein bands were observed with an enhanced chemiluminescence kit.

#### 2.7.3 RNA interference and cell transfection

SiRNA-INSR and siRNA-control were designed and synthesized by GenePharma Biologicals (Shanghai, China). The sequences are as follows: 5’- GGAUCACGACUGUUCUUUATT-3’ (siRNA-INSR); 5’- AAUUCUCCGAACGUGUCACTT-3’ (siRNA-control). Then, according to the instructions, MG-63 cells were transfected with Lipofectamine 3000 reagent (Invitrogen, Carlsbad, CA, USA), and siRNA-con transfection was used as a control. After 72h, the expression of INSR in MG-63 cells was detected by WB.

#### 2.7.4 Cell transwell assay

As described previously (13), simply add 500 uL of complete medium to the lower chamber of the Transwell pre-coated with Matrigel matrix and 150 uL of MG-63 cell suspension to the upper room and incubate in the incubator. 12 h later, remove the chambers, wash once with PBS (gently), then place the sections in a 24-well plate (600 μL of 4% paraformaldehyde) and fix for 20-30 min and wash twice with PBS. Next, the chambers were stained in crystalline violet staining solution (600 μL 0.1%) for 10-20 min and washed twice with PBS. Finally, the cells inside the chambers were wiped with cotton swabs and air-dried, microscopically observed the chambers’ membranes.

### 2.8 Statistical analysis

All the data in our study were analyzed by R version4.0.2 (http://www.R-project.org). Univariate Cox regression analysis was used to assess the correlation between gene expression and patient prognosis. Multivariate Cox regression analysis was used to establish risk profiles based on genes associated with prognosis in patients with OS and analysis of differences in survival between high- and low-risk groups generated by log-ranking tests defined by K-M analysis. All cell experiments were repeated three times, and the differences between data were analyzed for significance by Student`s t-test. P<0.05 was considered significant in all statistical tests.

## 3 Results

### 3.1 Acquisition of glycolysis pathway gene set and screening of differential genes

**Figure 2:**
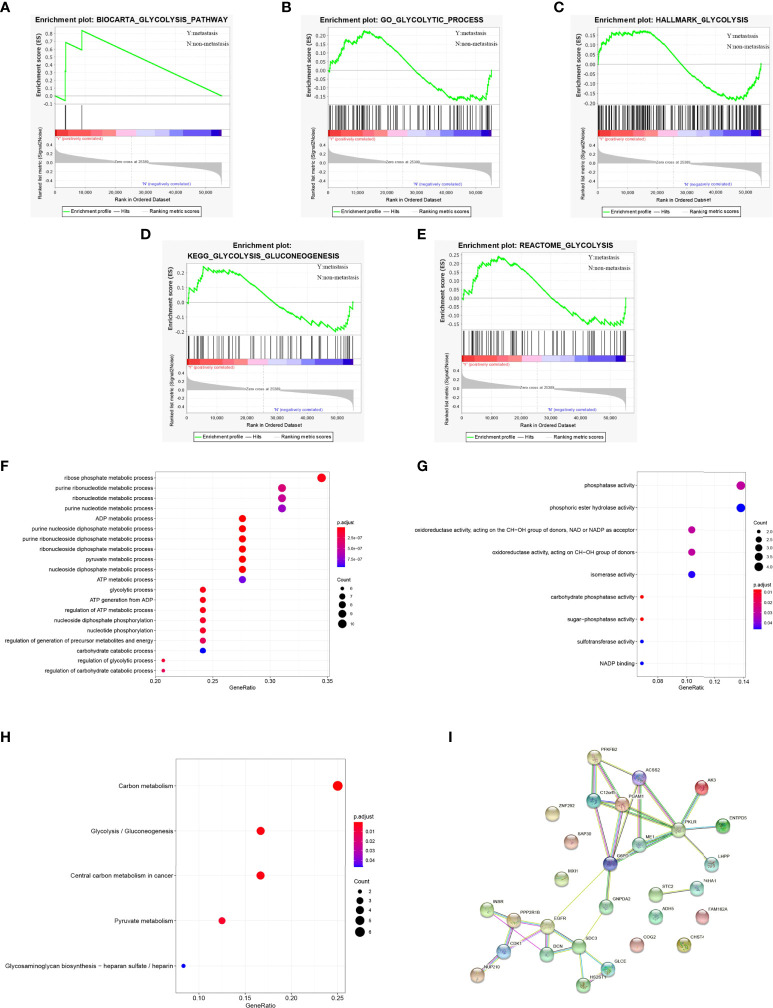
GSEA identified that five glycolysis gene sets were significantly enriched. **(A)** BIOCARTA GLYCOLYSIS PATHWAY. **(B)** GO GLYCOLYTIC PROCES. **(C)** HALLMARK GLYCOLYSIS. **(D)** KEGG GLYCOLYSIS GLUCONEOGENESIS. **(E)** REACTOME GLYCOLYSIS. **(F)** GO-BP analysis of 29 survival-related genes. **(G)** GO-MF analysis of 29 survival-related genes. **(H)** KEGG analysis of 29 survival-related genes. **(I)** PPI network diagram of 29 survival-related genes.

We obtained 5 glycolysis-related pathway gene sets from MSigDB, namely BIOCARTA GLYCOLYSIS PATHWAY, GO GLYCOLYTIC PROCES, HALLMARK GLYCOLYSIS, KEGG GLYCOLYSIS GLUCONEOGENESIS, REACTOME GLYCOLYSIS. The GSEA results of the OS dataset from the TARGET database show that five glycolysis-related pathway gene sets are significantly different in patients with metastatic and non-metastatic OS (P<0.05, **Figures 2A–E**).

### 3.2 Screening of genes related to OS prognosis

We extracted 326 genes from the 5 glycolysis-related pathway gene sets. Next, use R language to sort out the gene sequencing data of OS patients from the TARGET database and extract a total of 326 human glycolysis-related gene expression profiles. The results of univariate Cox regression analysis showed that 29 glycolytic pathway-related genes were significantly associated with the overall survival of OS patients (P<0.05, **Supplementary Table 1**).

### 3.3 GO and KEGG pathway analysis

For 29 prognostic-related genes, the results of GO analysis show that these glycolytic genes can be enriched in some basic biological processes. It includes ADP metabolism, pyruvate metabolism, purine nucleoside diphosphate metabolism and nucleoside diphosphate metabolism (**Figure 2F**). Some of them are enriched in molecular functions, which affect carbohydrate phosphatase activity, sugar phosphatase activity, phosphatase activity, oxidoreductase activity and so on (**Figure 2G**). Meanwhile, KEGG analysis showed that these 29 prognostic genes were mainly related to carbon metabolism, glycolysis/gluconeogenesis, pyruvate metabolism, glycosaminoglycan biosynthesis, heparan sulfate/heparin (**Figure 2H**). These genes are linked to form a PPI network (**Figure 2I**).

### 3.4 Construction of risk model for OS prognosis

**Figure 3:**
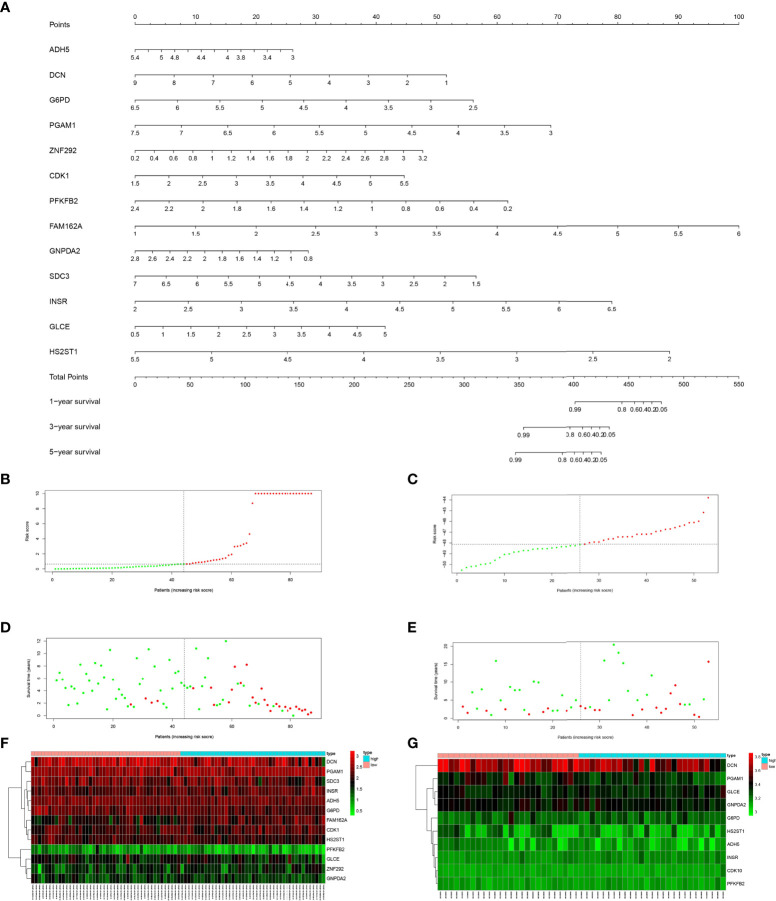
**(A)** Nomogram of prediction model for OS patients. **(B)** Risk score distribution of low-risk (green) and high-risk (red) in TARGET patients with OS. **(C)** Risk score distribution of low-risk (green) and high-risk (red) in GSE21257 patients. **(D)** Scatter plot of survival status of TARGET patients with OS. Red dots (dead); green dots (alive). **(E)** Scatter plot of survival status of GSE21257 patients in. Red dots (dead); green dots (alive). **(F)** Expression of risk genes in the high-risk (blue) and low-risk (pink) of TARGET patients with OS. **(G)** Expression of risk genes in the high-risk (blue) and low-risk (pink) of GSE21257 patients with OS.

For these 29 prognosis-related genes, we finally included 13 genes (ADH5、DCN、G6PD、PGAM1, ZNF292, CDK1, PFKFB2, FAM162A, GNPDA2, SDC3, INSR, GLC, HS2ST1) through multivariate Cox regression analysis to construct the prognosis risk model (**Table 2**) and draw the nomogram (**Figure 3A**). The risk score of the constructed prognostic risk model=(ADH5×-0.795)+(DCN×-0.470)+(G6PD×-1.020)+(PGAM1×1.114)+(ZNF292×1.157)+(CDK1×0.812)+(PFKFB2×2.043)+(FAM162A×1.456)+(GNPDA2×-1.047)+(SDC3×-0.748)+(INSR×1.278)+(GLCE×0.671)+(HS2ST1×-1.841). See **Supplementary Table 2** for the results of multivariate Cox regression analysis.

**Table 2:** Characteristics of genes in the prognostic model.

Gene	Univariate analysis			Multivariate analysis			Coefficients
	HR	95%CI	P-value	HR	95%CI	P-value	
ADH5	0.397	0.178-0.885	0.024	0.452	0.162-1.259	0.129	-0.795
DCN	0.657	0.496-0.869	0.003	0.625	0.427-0.915	0.016	-0.470
G6PD	0.369	0.196-0.693	0.002	0.361	0.162-0.803	0.013	-1.020
PGAM1	1.652	1.0416-2.620	0.033	0.328	0.124-0.869	0.025	-1.114
ZNF292	2.168	1.033-4.551	0.041	3.182	1.167-8.679	0.024	1.157
CDK1	1.830	1.079-3.103	0.025	2.253	1.154-4.398	0.017	0.812
PFKFB2	0.194	0.0496-0.755	0.018	0.130	0.026-0.640	0.012	-2.043
FAM162A	1.832	1.259-2.667	0.002	4.288	2.110-8.711	5.69E-05	1.456
GNPDA2	0.384	0.158-0.932	0.034	0.351	0.087-1.419	0.142	-1.047
SDC3	0.585	0.399-0.859	0.006	0.473	0.268-0.835	0.010	-0.748
INSR	1.982	1.124-3.493	0.018	3.590	1.702-7.574	0.0008	1.278
GLCE	0.350	0.164-0.746	0.006	1.956	0.831-4.605	0.124	0.671
HS2ST1	0.439	0.204-0.944	0.035	0.159	0.045-0.559	0.004	-1.841

### 3.5 Evaluation and validation of prognostic risk models

As shown in **Figures 3B, C**, we sorted the risk scores of all OS patients to get the distribution of the survival rate. From the scatter plot, we found that with the increase in risk score, the mortality of patients increased gradually (**Figures 3D, E**). After calculation, the genes with Hazard Ratio (HR) > 1 (ZNF292, CDK1, FAM162A, INSR, GLCE) were classified as risk genes, while the genes with HR < 1 (ADH5, DCN, G6PD, PGAM1, PFKFB2, GNPDA2, SDC3, HS2ST1) were protective genes. Patients in the high-risk group are more likely to express risk genes, while patients in the low-risk group tend to express protective genes (**Figures 3F, G**).

**Figure 4:**
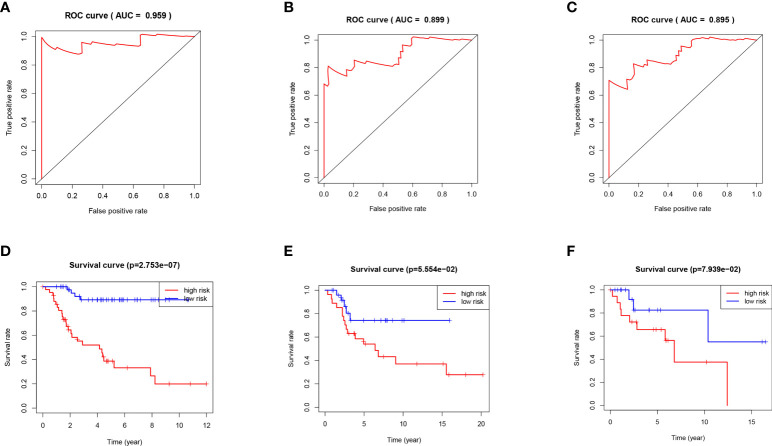
**(A)** ROC curve analysis of 1 year survival in TARGET patients with OS. **(B)** ROC curve analysis of 3 years survival in TARGET patients with OS. **(C)** ROC curve analysis of 5 years survival in TARGET patients with OS. **(D)** K-M curve of TARGET patients with OS. **(E)** K-M curve of GSE21257 patients with OS. **(F)** K-M curve of GSE39055 patients with OS.

Next, the area under the curve (AUC) of 1-year, 3-years and 5-years survival rates of OS patients were 0.959, 0.899 and 0.895, respectively, which means that the risk model has a significant prognostic value (**Figures 4A–C**). Furthermore, according to the K-M curve drawn by the prognosis model, the survival rates of the TARGET osteosarcoma data set, GSE21257 data set and GSEGSE39055 data set in the high-risk group were significantly lower than those in the low-risk group (P<0.05, **Figures 4D–F**).

### 3.6 Further screening for metastasis-associated prognostic genes

**Figure 5:**
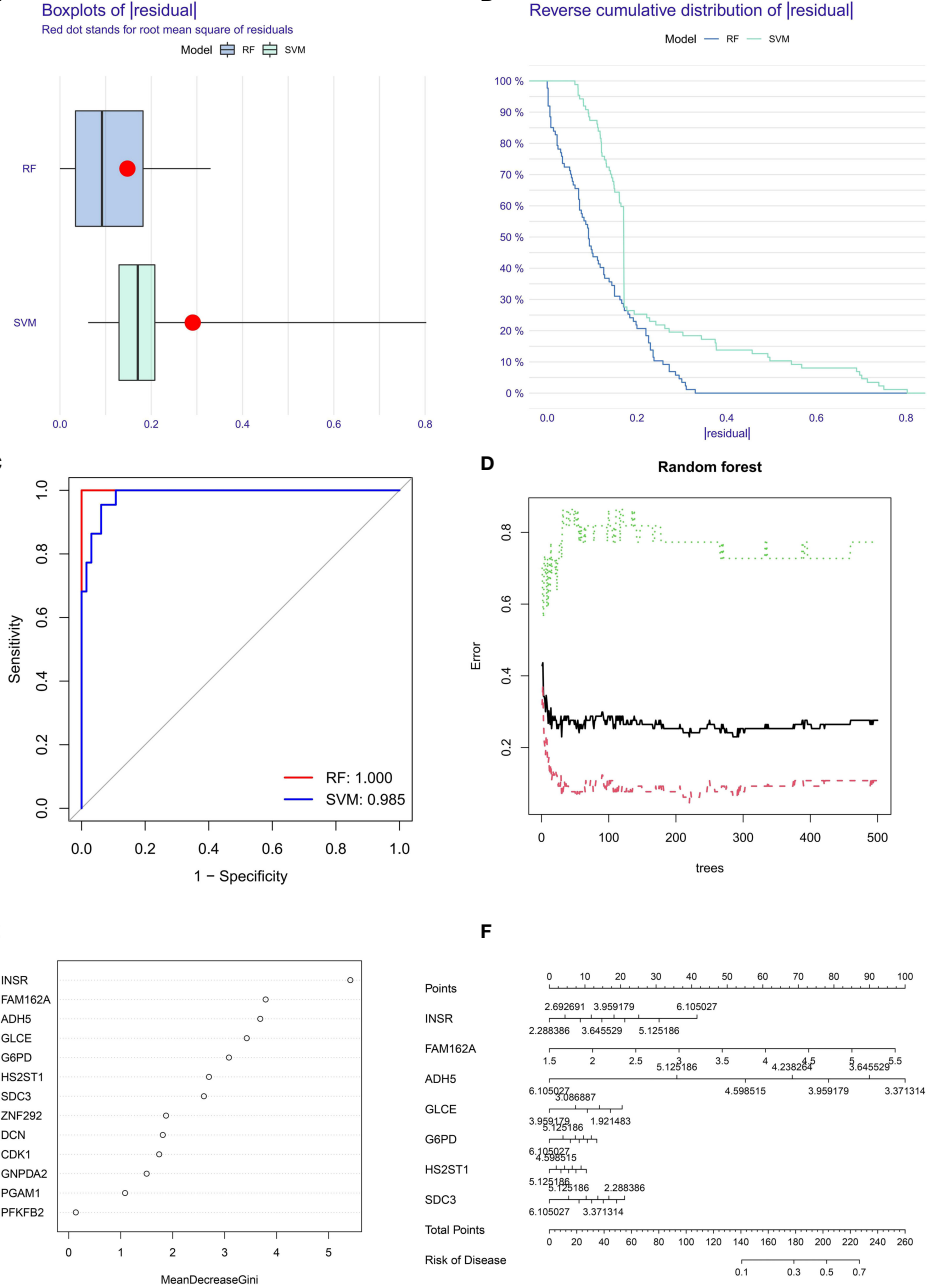
Box plots **(A)** and error analysis **(B)** of two unsupervised clustering methods for OS-based differential expression of metastasis and non-metastasis genes, and comparison of the accuracy **(C)** of the two operational models, with the RF model **(D)** classifier iteration process. **(E)** The order of importance of the correlation between prognostic genes and tumor metastasis. **(F)** Seven prognostic genes with the highest correlation with OS metastasis constructed a metastasis related disease model.

Sample classification models were constructed using SVM and RF based on optimal feature prognosis gene combinations to construct SVM and RF classifiers based on OS differential expression values of metastasis and non-metastasis groups and unsupervised clustering methods for error analysis in the TARGET dataset (**Figures 5A, B**). The results showed that the best prognostic gene combination had the highest accuracy in categorical metastasis when the prognostic number was set to 5. As shown in **Figure 5C**, the RF constructed sample classification model has higher accuracy (AUC is close to 1) compared to SVM (AUC = 0.985).

The RF classifier’s iterative calculation process was shown in **Figure 5D**. The RF classifier algorithm obtained the specific importance ranking of prognostic genes in terms of tumor metastasis correlation (**Figure 5E**) and finally screened the seven prognostic genes with the highest correlation with tumor metastasis (INSR, FAM162A, GLCE, ADH5, G6PD, SDC3, HS2ST1), and constructed metastasis-related disease models based on the seven genes (**Figure 5F**).

### 3.7 *In vitro* experimental validation

**Figure 6:**
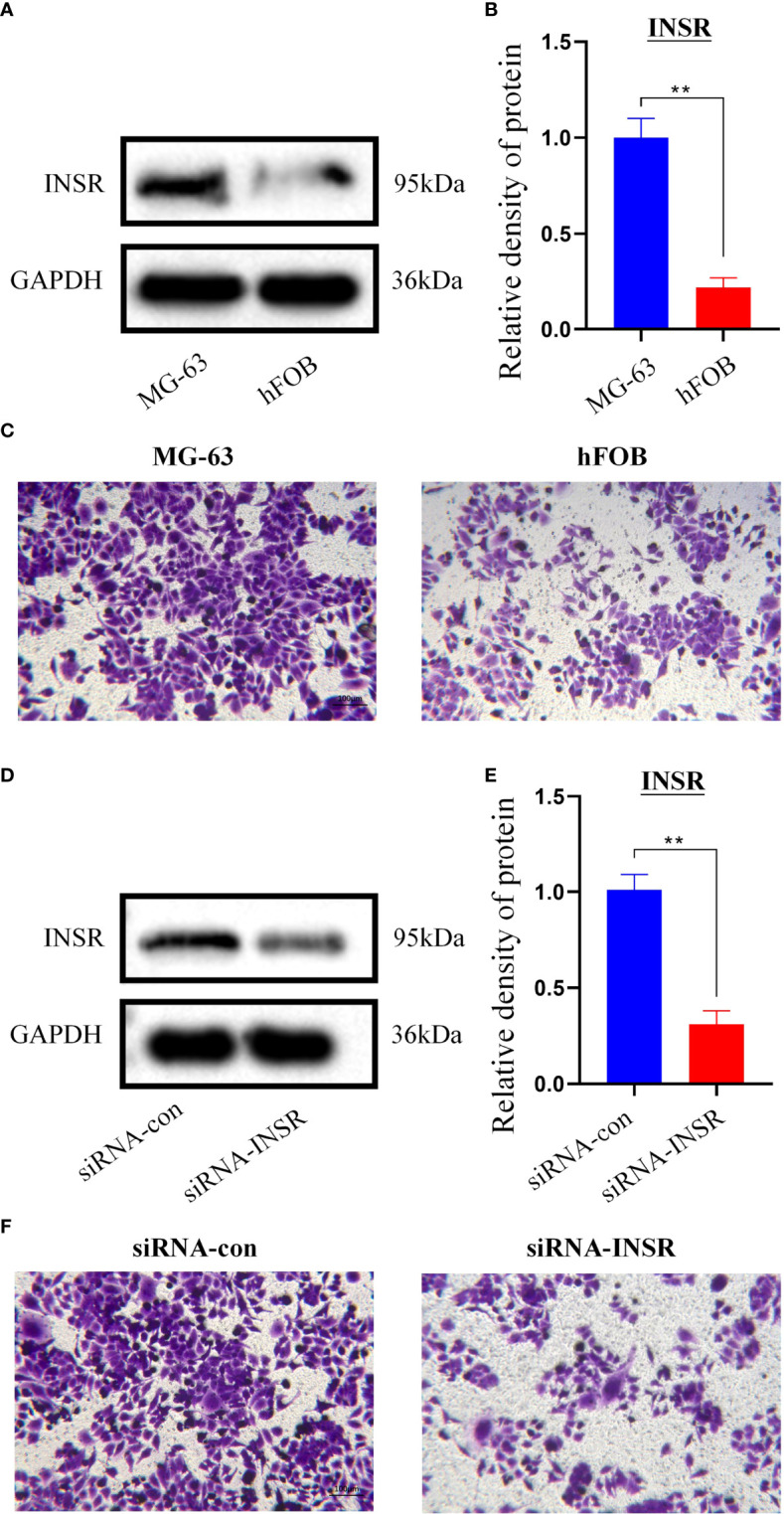
*In vitro* validation of INSR gene. **(A)** Expression of INSR protein by western blotting in MG-63 cells and hFOB 1.19 cells. **(B)** Relative density of INSR protein expression in MG-63 cells and hFOB 1.19 cells. n = 3, data are represented as mean ± SD, **P < 0.01 vs. MG-63 cells. **(C)** Transwell invasion of MG-63 cells and hFOB 1.19 cells. **(D)** Expression of INSR protein by western blotting in MG-63 cells after transfection of siRNA-control and siRNA-INSR. **(E)** Relative density of INSR protein expression in MG-63 cells after transfection of siRNA-control and siRNA-INSR. n = 3, data are represented as mean ± SD, **P < 0.01 vs. siRNA-control. **(F)** Transwell invasion of MG-63 cells after transfection of siRNA-control and siRNA-INSR.

The INSR gene, which has the highest importance with OS metastasis, was finally selected for validation. The WB results showed that INSR protein expression was higher in human MG-63 osteosarcoma cells than in human hFOB1.19 osteoblasts (P<0.01, **Figures 6A, B**). Also, Transwell assays confirmed that MG-63 cells migrated more significantly than hFOB1.19 cells (P<0.01, **Figure 6C**). To further verify the function of the INSR gene in OS invasion, we designed and synthesized siRNA-INSR targeting the INSR gene and transfected it into MG-63 cells with Lipo3000, using siRNA-control as control. Subsequent WB results showed that siRNA-INSR effectively reduced INSR protein expression (P<0.01, **Figures 6D, E**). Transwell assays also showed a significant decrease in invasion of MG-63 cells after siRNA-INSR transfection compared to the control group (P<0.01, **Figure 6F**). These results further confirmed the important regulatory role of INSR on OS metastasis.

## 4 Discussions

Osteosarcoma is highly invasive and metastatic, often leading to a poor prognosis (14, 15). Therefore, it is urgent to find effective biomarkers for OS-specific forecasts to improve the management of OS patients. Considering the importance of the glycolysis pathway in the occurrence and progression of cancer (16, 17), it is necessary to search for glycolysis pathway-related biomarkers for the prognosis of OS patients.

In this study, we constructed a prognostic risk model of OS based on 13 key genes in glycolysis pathways and improved the prognostic ability of OS patients at the gene level. The results show that the risk model can successfully divide OS patients into high-risk and low-risk groups, significantly affecting overall survival.

Alcohol dehydrogenase5 (ADH5) is an important formaldehyde catabolism enzyme, and malignant tumor cells often produce a large amount of by-products-endogenous formaldehyde during their physiological processes (18). Therefore, the activity of adh5 is considered an excellent prognostic marker by oncologists. Studies have shown that ADH5 and ADH7 may play an anti-tumor role in the carcinogenesis of Non-small cell lung cancer (NSCLC) and can be used as biomarkers to predict NSCLC patients (19). At the same time, ADH5 is also considered an important tumor suppressor factor for gastric cancer (18). Our research has also confirmed that the level of ADH5 can indeed affect the survival time of OS patients. Decorin (DCN) is a multi-functional molecule of the extracellular matrix. It is considered a natural tumor suppressor, inhibiting the growth and metastasis of various cancer cells *in vitro* (16). DCN can not only inhibit osteosarcoma cell-mediated angiogenesis (20), but also the lung metastasis of osteosarcoma in mice (21).

Glucose 6-phosphate dehydrogenase (G6PD) is an important enzyme that assists glucose metabolism. Studies have confirmed that LncRNA OR3A4 can regulate the growth of osteosarcoma cells by regulating miR-1207-5p/G6PD signaling, and inhibition of OR3A4 can increase the expression of miR-1207-5p, thereby inhibiting the level of G6PD in OS cells (22). The low level of G6PD inhibits the levels of NADPH and glucose in osteosarcoma cells. Consumption and lactic acid production, thereby preventing the progression of OS. Phosphoglycerate mutant enzyme-1 (PGAM1) is a glycolytic gene that can promote the conversion of 3-phosphoglycerate to 2-phosphoglycerate to glycolysis, which can promote cancer cell proliferation and survival (23). Therefore, the level of PGAM1 seems to predict the prognosis of various tumors, such as lung cancer, breast cancer, lymphoma and so on (24–26).

ZNF292 is considered to be a potential suppressor gene (TSG) of gastrointestinal cancer (gastric cancer, liver cancer and colorectal cancer) (27). Studies have confirmed that Circular RNA ZNF292 can affect the proliferation and apoptosis of liver cancer cells by regulating the Wnt/β-catenin pathway, so knocking down circRNA ZNF292 can cause cancer cell cycle to arrest in the G1 phase, thereby inhibiting cell proliferation and promoting the cell apoptosis (28). Similarly, Cyclclindependent kinase 1 (CDK1) belongs to the serine/threonine protein kinase family and is closely related to cell cycle and growth (29). Studies have confirmed that reducing CDKI activity can induce cycle arrest and apoptosis of osteosarcoma cells, thereby reducing the survival of osteosarcoma cells (30).

Tumor cells generally increase glucose metabolism through glycolysis and pentose phosphate pathways to meet rapid cell proliferation’s bioenergy and biosynthesis requirements, and 6-phosphofructo-2-kinase (PFKFB2) is a key enzyme for glycolysis (31). Studies have confirmed that miR-1297 can inhibit osteosarcoma’s proliferation and aerobic glycolysis by regulating PFKFB2 (32). Studies have also found that the SLIT2/ROBO1 axis promotes the Warburg effect of osteosarcoma by activating the SRC/ERK/c-MYC/PFKFB2 pathway (33). Therefore, PFKFB2 is a key factor in the development of osteosarcoma and is closely related to the prognosis of osteosarcoma.

The syndecan (SDC) family consists of four cell surface molecular members (SDC-1 to 4) with different biological functions. Among them, SDC-3 is mainly expressed in brain and nerve tissues and plays a key role in cell development, adhesion and migration (34). Studies have shown that hypoxia can promote the expression of Syndecan-3 so that patients with melanoma tumors have a better overall survival rate (35). Heparan sulfate 2-O-sulfotransferase 1 (HS2ST1) is one of several special enzymes required for synthesizing heparan sulfate, which can catalyze the metastasis of sulfate radical to the sugar moiety of heparan sulfate (36). HS2ST1 plays an essential role in the migration and differentiation of malignant tumors (37). Previous studies have confirmed that the HS2ST1-dependent signal transduction pathway determines the cell viability, matrix interaction and invasive behavior of breast cancer. The increase of HS2ST1 expression significantly promotes the invasiveness of breast cancer cells, leading to a poor prognosis (38).

Insulin receptor (INSR) and IGF1R are both tyrosine kinase receptors (RTKs) and belong to the insulin-like growth factor system (IGFs). The IGFs family mainly consists of IGF1R, IGF2R, INSR, INSR/IGF1R, INSR-related Receptor (IRR) and IGFBP-1~7, which have the function of regulating cell growth and energy metabolism, and have the function of promoting cell proliferation, migration and differentiation (39, 40). The INSR gene encodes a transmembrane tetrameric receptor protein, the insulin receptor. INSR is mainly expressed in tissue targets of insulin metabolic effects: liver, adipose tissue and skeletal muscle, and mainly receives signals from extracellular insulin, thus ensuring insulin metabolic levels in adult individuals (41). In addition, ISNR is also overexpressed in many tumors, colon, breast, lung, and thyroid cancers, among others (42, 43). Our study also found high expression of INSR in MG-63 osteosarcoma cells, which correlates with high levels of glycolysis in tumor cells.

Studies have also shown that the expression of SDC-3 protein has an excellent predictive value for the prognosis of breast cancer (44). INSR can bind to ligands such as insulin and insulin-like growth factor (IGF-1 or IGF-2) to change the structure and conformation, activate intracellular tyrosine kinase, and initiate intracellular signal transduction. Play important physiological functions in the organism (45). INSR is closely related to the metastasis of osteosarcoma. Early studies have shown that blocking the IGF/IGF-1R signal axis with soluble IGF-1R mutants can inhibit the cell proliferation and tumor growth of human osteosarcoma (46). At the same time, MiRNA-466 can also inhibit cell proliferation and invasion in osteosarcoma by directly targeting insulin receptor substrate 1 (47).

Although the clinical significance of glycolysis-related gene signatures in predicting metastasis and prognosis in patients with osteosarcoma has been well established, genome-wide analyses to identify the specific underlying molecular mechanisms of glycolysis-related gene signatures in osteosarcoma are currently lacking, especially with the advent of liquid biopsies and next-generation sequencing. Combined analysis of DNA/RNA and metabolic biomarkers in osteosarcoma or other bone tumors may further elucidate the key steps of glycolysis in regulating the metabolism of tumor cells.

## 5 Conclusions

We developed a new prognostic risk model for osteosarcoma based on 7 glycolytic genes (INSR, FAM162A, GLCE, ADH5, G6PD, SDC3, HS2ST1) by bioinformatics. We screened INSR as the gene with the highest importance with OS metastasis by machine learning, and *in vitro* experiments also confirmed the effect of INSR on OS migration with a vital facilitation role. These glycolytic metabolic pathway genes also open new possibilities for targeted therapy in osteosarcoma.

## Data availability statement

The datasets presented in this study can be found in online repositories. The names of the repository/repositories and accession number(s) can be found in the article/**Supplementary Material**.

## Author contributions

WH carried out the acquisition and interpretation of data and was the major contributor to drafting the manuscript. YX was responsible for data statistics and analysis. HW and KL were responsible for *in vitro* validation. GC participated in drawing tables and diagrams. YX and HW contributed to the article’s ideas and reviewed the manuscript. All authors provided final approval for publishing the manuscript.

## Funding

In vitro experimental validation of this study was supported by Special Fund for Scientific Research ("520" Talent Project) of Dongguan Tungwah Hospital (YJY202102).

## Acknowledgments

We are grateful to the Home for Researchers (www.home-for-researchers.com) for its support for my manuscript’s language expression and grammar.

## Conflict of interest

The authors declare that the research was conducted in the absence of any commercial or financial relationships that could be construed as a potential conflict of interest.

## Publisher’s note

All claims expressed in this article are solely those of the authors and do not necessarily represent those of their affiliated organizations, or those of the publisher, the editors and the reviewers. Any product that may be evaluated in this article, or claim that may be made by its manufacturer, is not guaranteed or endorsed by the publisher.

## Supplementary material

Supplementary Table 1 Univariate Cox regression analysis.

Supplementary Table 2 Multivariate Cox regression analysis.
